# National Trends in Rectal Cancer Among Younger Patients: A National Cancer Database Analysis

**DOI:** 10.7759/cureus.107699

**Published:** 2026-04-25

**Authors:** Salma Khan, Nolberto Jaramillo, Adrianus J Ekelmans, David J Samson, Mikaiel J Ebanks, Samantha Norden, Ryan Bendl, James Clarke, Madalyn G Neuwirth

**Affiliations:** 1 Surgery, Westchester Medical Center, Valhalla, USA

**Keywords:** colorectal cancer, national cancer database and seer analyses, rectal cancer, tumor grade, young onset colorectal cancer

## Abstract

Introduction: In recent years, the incidence of new rectal cancer diagnoses among younger patients (< 50 years) has been increasing at alarming rates. Paradoxically, previous studies show that younger patients present with more advanced-stage disease; however, they have better survival rates than older individuals. In this study, we aim to examine the clinical and pathologic trends in younger patients with rectal cancer in a contemporary cohort.

Methods: A retrospective cohort study of the National Cancer Database (NCDB) was conducted to examine characteristics of invasive rectal cancer diagnoses between younger (< 50 years old) and older (≥ 50 years old) individuals in a dataset from 2004 to 2021. Joinpoint regression analysis (version 5.3.0.0, U.S. National Cancer Institute, Bethesda, MD, USA) was used to examine trends in rectal cancer over time. Chi-square analyses were utilized to determine differences in proportions of patients with earlier (Stage I/II) and later (Stage III/IV) clinical stage or grade disease between patients in the two age groups. Overall survival (all-cause mortality) was compared between groups using Kaplan-Meier survival analysis. P-values < 0.05 were considered statistically significant.

Results: A total of 171,543 rectal adenocarcinoma patients from NCDB were analyzed. Joinpoint analysis revealed a rise in rectal cancer rates for both older (annual percent change (APC) 4.2% from 2011 to 2021) and younger groups (5.4% from 2004 to 2021), with a more rapid increase in the younger group. Younger patients were more likely to present with advanced stage (III and IV) disease (p < 0.001) and higher-grade tumors (p < 0.001). Kaplan-Meier survival analysis showed worse median survival for younger patients at advanced (III and IV) stages of disease (-0.129 years, p = 0.016); however, there was no difference at earlier stages (p = 0.900). When stratified by tumor grade, those with higher-grade (Grade 3/4) tumors had significantly better survival than those with lower-grade (Grade 1/2) tumors (median difference: -1.071 years, p < 0.001).

Conclusion: There is a rising incidence of rectal cancer in young patients, who often present with advanced disease and demonstrate worse outcomes than older patients when advanced, likely due to delayed diagnosis and treatment. The high prevalence of late-stage diagnoses underscores the need for improved awareness and targeted screening strategies to promote earlier detection. Addressing these disparities is essential to optimizing screening, care, and outcomes for these patients.

## Introduction

Colorectal cancer represents the third most common cancer and the third leading cause of cancer-related death worldwide [1]. Although the overall incidence of colorectal cancer has decreased owing to the implementation of population-based screening, there has been an alarming increase in the incidence of rectal cancer among young adults under the age of 50 years [1, 2]. It is estimated that by 2030, nearly one in four rectal cancers will be diagnosed in individuals aged less than 50 years [1]. The reasons for this disproportionate increase are unknown. Although young-onset disease may arise in the context of a hereditary cancer syndrome, the majority of cases are sporadic, with considerable genotypic and phenotypic heterogeneity [3, 4].

Due to the rising incidence of colorectal cancer in younger individuals, guidelines from the American Cancer Society (ACS) and the United States Preventive Services Task Force (USPSTF) now recommend starting routine screening at 45 instead of 50 for average-risk individuals. Those with a strong family history or genetic predispositions may require screening as early as 20-25 years. These changes aim to enhance early detection and intervention to mitigate the growing disease burden [5].

Surveillance, Epidemiology, and End Results (SEER) data indicate that younger patients are more likely to present with advanced-stage disease, with nearly 50% diagnosed at stage III or IV, compared to 30% of older adults. Similarly, National Cancer Database (NCDB) findings show a higher prevalence of locally advanced and metastatic disease in younger individuals [6]. Despite this aggressive presentation, younger patients have comparable or slightly better survival rates, with five-year survival for localized rectal cancer exceeding 90%, compared to 85% in older cohorts [7]. This paradox, a more advanced disease but improved survival, highlights the need for a deeper understanding of rectal cancer in younger patients, emphasizing prevention, early detection, and personalized treatment approaches. Additionally, whether age at presentation serves as an independent adverse prognostic factor remains uncertain. This study aims to analyze the clinical and pathological characteristics of rectal cancer in younger patients, examining diagnostic trends over a contemporary multi-decade period.

## Materials and methods

This study utilized the NCDB (2004-2021), a quality improvement and surveillance initiative conducted in collaboration with the American College of Surgeons (ACS). The NCDB collects data from accredited centers to analyze patient demographics and clinical outcomes, improving cancer care. The NCDB is a joint project of the Commission on Cancer of the ACS and the American Cancer Society. The study protocol was approved by the Institutional Review Board of Westchester Medical Center, Valhalla, NY, USA (IRB #23992) and deemed exempt, as all NCDB data are de-identified, and written consent was not required for this retrospective analysis.

Patients diagnosed with rectal adenocarcinoma were identified using the following International Classification of Diseases for Oncology, Third Edition (ICD-O-3) histology codes: 8140, 8144, 8213, 8220, 8255, 8262, 8480, and 8481 [8]. Only invasive cancers (behavior code 3) were included, and patients with missing tumor grade or clinical stage data were excluded. Tumor grade was categorized as Grade 1 & 2 (well/moderately differentiated) or Grade 3 & 4 (poorly/undifferentiated), while clinical stage was classified as Stage I & II (early stage) or Stage III & IV (advanced stage). Patients were stratified into two age groups: < 50 years (younger-onset) and ≥ 50 years (older-onset).

A retrospective cohort analysis was conducted to compare tumor grade, stage, and survival outcomes between age groups. Chi-square tests were used to assess differences in tumor grade and stage distributions. Kaplan-Meier survival analysis was performed to compare overall survival, and differences between groups were evaluated using the log-rank test. Joinpoint regression analysis (version 5.3.0.0, U.S. National Cancer Institute, Bethesda, MD, USA) was used to examine trends in rectal cancer incidence over time, identifying statistically significant changes and calculating the annual percent change (APC) for each segment. The trend analysis was performed separately for patients < 50 years and ≥ 50 years. All statistical analyses were conducted using IBM SPSS Statistics software, version 29 (IBM Corp., Armonk, NY, USA), with statistical significance set at p < 0.05. 

To control for potential confounding in the comparison between tumor grade groups, we used propensity score matching. A propensity score model was constructed using multivariable logistic regression. The dependent variable was whether patients had a poorly or undifferentiated tumor grade, in contrast with a well- or moderately differentiated tumor grade. Input independent variables to the model included age, sex, race, stage, Charlson-Deyo Comorbidity Score, chemotherapy, immunotherapy, regional lymph node surgery, surgical procedure type at primary site, surgery of other site, surgical margins status, radiation-surgery sequence, systemic therapy-surgery sequence, and year of diagnosis. Propensity scores were predicted probabilities of the logistic regression model. Patients with poorly or undifferentiated tumour grades were considered cases, and those with well or moderately differentiated tumor grades were considered controls. Cases were matched with controls on a 1:1 basis with a maximum tolerance of 0.02. Between-group balance on patient characteristics was assessed with absolute standardized mean differences (ASMDs). An ASMD < 0.10 was considered a good balance, and values between 0.11 and < 0.20 were considered an adequate or acceptable balance.

## Results

Out of the total sample of 171,543 patients diagnosed with rectal adenocarcinoma, all had complete data on tumor grade and clinical stage. When stratified by age group, patients under 50 years of age accounted for 28,898 (16.8%) of the sample, while those aged ≥ 50 years comprised 142,645 (83.2%) (Table 1). 

**Table 1:** Participant sample flow numbers A total of 171,543 patients had completed data and were included in the analysis. NCDB: National Cancer Database; ICD-O-3: International Classification of Diseases for Oncology, Third Edition

	Description	No. (% of original)	No. excluded from previous
1	NCDB cancer of the rectum, 2004-2021 (all)	413,018	
2	Limited to ICD-O-3 histology codes: 8140, 8144, 8213, 8220, 8255, 8262, 8480, 8481	301,733 (73.1%)	111,285
3	Limited to behavior code 3 (invasive)	298,087 (72.2%)	3,646
4	Limited to stage group not missing AND grade not missing	171,543 (41.5%)	126,544

Younger patients were more likely to present with advanced-stage disease (Stage III/IV), with 67.3% of patients under 50 falling into this category compared to 53.4% of those aged ≥50 (p < 0.001) (Table 2). Conversely, older patients were more likely to be diagnosed with early-stage disease (Stage I/II) (46.6% vs. 32.7%, p < 0.001). Additionally, younger patients were slightly more likely to have high-grade tumors (Grade 3/4), with 14.3% of patients under 50 diagnosed with poorly or undifferentiated adenocarcinoma, compared to 12.8% in the older cohort (p < 0.001) (Table 2). 

**Table 2:** Association between age and clinical stage group and association between age and tumor grade

Variable	Subgroup	Age < 50 years vs 50+ years				p-value
		< 50		50+		
		No.	%	No.	%	
Clinical stage group	I/II	9,464	32.7%	66,522	46.6%	<0.001
	III/IV	19,434	67.3%	76,123	53.4%	<0.001
Tumor grade	1/2	24,760	85.7%	124,365	87.2%	<0.001
	3/4	4,138	14.3%	18,280	12.8%	<0.001

**Figure 1:**
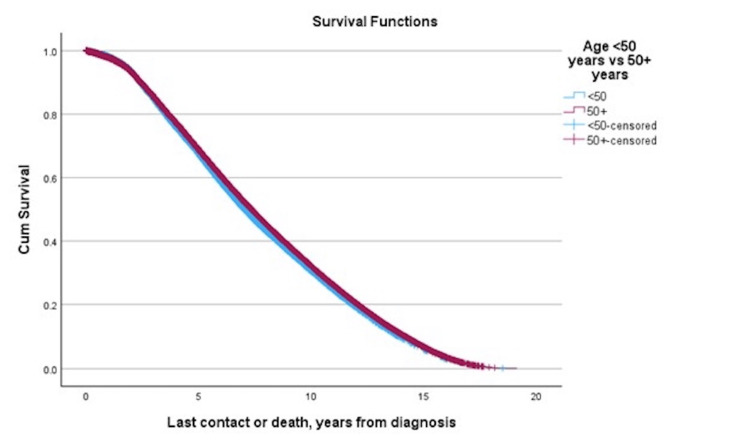
Survival curve demonstrating overall survival by age.

Kaplan-Meier survival analysis revealed that younger patients had significantly worse overall survival compared to those aged 50+ (median difference: -0.320 years, p < 0.001) (Figure 1, Table 3).

**Table 3:** Median overall survival by age group.

Age < 50 years vs 50+ years		Row med (years)	50+ col med (years)	Δ	p-value
Log rank	<50	7.009	7.329	-0.320	<0.001

**Figure 2:**
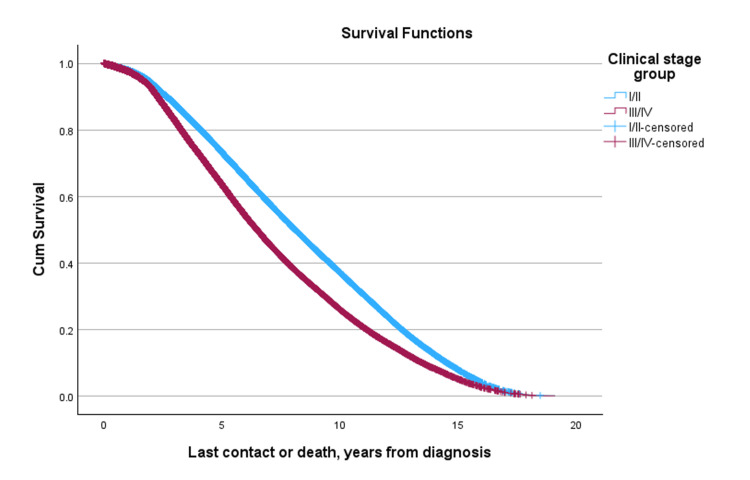
Cumulative survival curves by clinical stage group.

Median survival by stage was significantly different across all groups, with Stage IV patients exhibiting the lowest median survival years (Table 4, Figure 2). 

**Table 4:** Medial survival amongst all four stages.

	Row med (yrs)	Stage II col med (yrs)	Δ	p-value	Row med (yrs)	Stage III col med (yrs)	Δ	p-value	Row med (yrs)	Stage IV col med (yrs)	Δ	p-value
Stage I	8.006	8.140	-0.134	0.003	8.006	6.519	1.487	<0.001	8.006	6.232	1.774	<0.001
Stage II					8.140	6.519	1.621	<0.001	8.140	6.232	1.908	<0.001
Stage III									6.519	6.232	0.288	<0.001

**Figure 3:**
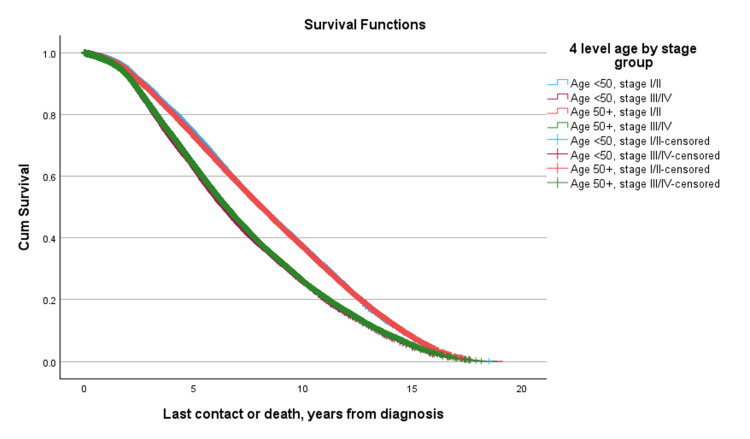
Overall survival by age and two-level clinical stage group

Median overall survival by combined stage and age group was as follows: for Stage I/II disease, survival was similar between patients aged ≥ 50 and < 50 (8.090 vs. 8.083 years, respectively); for Stage III/IV disease, older patients had higher median survival compared to younger patients (6.500 vs. 6.371 years, respectively). This survival disadvantage was more pronounced in patients with Stage III/IV disease, where younger patients exhibited lower median survival than their older counterparts (-0.129 years, p = 0.016) (Figure 3, Table 5). 

**Table 5:** Median overall survival by age and clinical stage group (young vs. old and stages I/II vs. III/IV).

Four-level age-by-stage group	Subgroup	Row med (years)	Age < 50, stage III/IV Col med (years)	Δ	P-value	Row med (yrs)	Age 50+, stage I/II Col med (yrs)	Δ	p-value	Row med (years)	Age 50+, stage III/IV Col med (years)	Δ	p-value
Log Rank	Age <50, Stage I/II	8.083	6.371	1.712	<0.001	8.083	8.090	-0.008	0.900	8.083	6.500	1.583	<0.001
	Age <50, Stage III/IV					6.371	8.090	-1.719	<0.001	6.371	6.500	-0.129	0.016
	Age 50+, Stage I/II									8.090	6.500	1.590	<0.001

**Figure 4:**
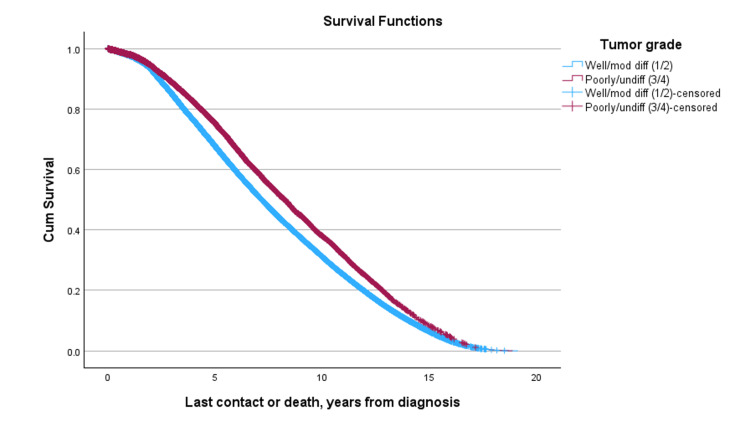
Survival curves by tumor grade, including well/moderately differentiated and poorly/undifferentiated tumors.

Median overall survival varied by both disease stage and patient age. In early-stage disease (Stages I and II), survival times were higher, with median survival ranging from 8.006 to 8.156 years across groups. Differences between younger patients (< 50 years) and older patients (≥ 50 years) were generally modest. For example, in patients with Stage I disease, the median difference in survival was minimal (Δ = -0.005 years, p = 0.081). Interestingly, Stage II was associated with slightly longer median survival compared to Stage I in both younger and older patients. Similarly, in Stage II disease, median survival remained high in both age groups, with a slight, non-significant advantage observed among younger patients (8.156 years vs. 8.143 years, Δ = +0.013 years, p = 0.148). In contrast, patients with advanced disease exhibited substantially shorter survival and pronounced differences by age. Median survival in patients with stage III and IV disease across age groups ranged from 6.048 to 6.538 years. Notably, younger patients did not experience a survival advantage in later-stage disease. In Stage III, younger patients had a slightly shorter median survival than older patients (Δ = -0.091 years, p = 0.038), while in Stage IV, median survival was also numerically lower in younger patients (Δ = -0.274 years), although this difference was not statistically significant (p = 0.243). Among patients diagnosed with Stage I/II disease, survival was similar between age groups (p = 0.900). When stratified by combined tumor grade, an unexpected survival trend was observed: patients with higher-grade (Grade 3/4) tumors had significantly better survival than those with lower-grade (Grade 1/2) tumors (median difference: -1.071 years, p < 0.001) (Figure 4, Table 6). 

**Table 6:** Median overall survival among well/moderately differentiated tumors (1/2), and poorly/undifferentiated tumors (3/4).

Tumor grade	Subgroup	Row med (years)	3/4 Col med (years)	Diff	p-value
Log Rank	1/2	7.168	8.238	-1.071	<0.001

**Figure 5:**
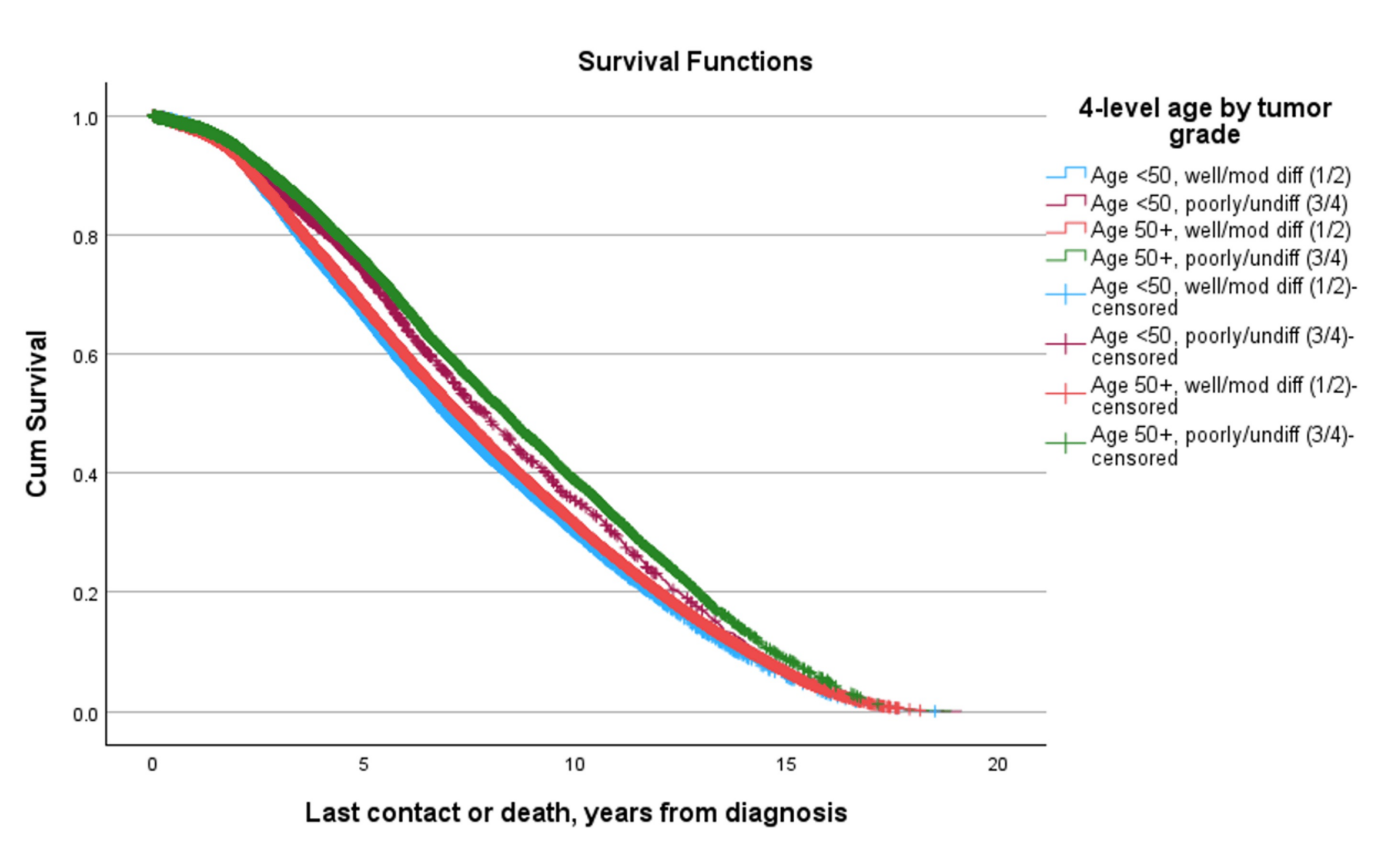
Cumulative survival curves for groups of combined age and tumor grade (<50 vs. >50 years, well/moderately differentiated vs. poorly/undifferentiated).

This counterintuitive finding persisted across both age groups, with younger patients with low-grade tumors (Grade 1/2) exhibiting the worst survival (-1.478 years, p < 0.001), compared to their older counterparts (Figure 5, Table 7). Among patients with high-grade tumors (Grade 3/4), those aged 50 and older had significantly better median overall survival compared to younger patients (8.685 vs. 8.386 years; Δ = 0.299, p < 0.001). 

**Table 7:** Median overall survival by age and two-level tumor grade

Four-level age-by-grade group	Subgroup	Row med (years)	Age < 50, grade 3/4 Col med (years)	Δ	p-value	Row med (years)	Age 50+, grade 1/2 Col med (years)	Δ	p-value	Age 50+, grade 3/4 Row med (years)	Col med (yrs)	Δ	p-value
Log Rank	Age < 50, grade 1/2	6.908	8.386	-1.478	<0.001	6.908	7.225	-0.318	<0.001	6.908	8.685	-1.777	<0.001
	Age < 50, grade 3/4					8.386	7.225	1.161	<0.001	8.386	8.685	-0.299	<0.001
	Age 50+, grade 1/2									7.225	8.685	-1.460	<0.001

**Figure 6:**
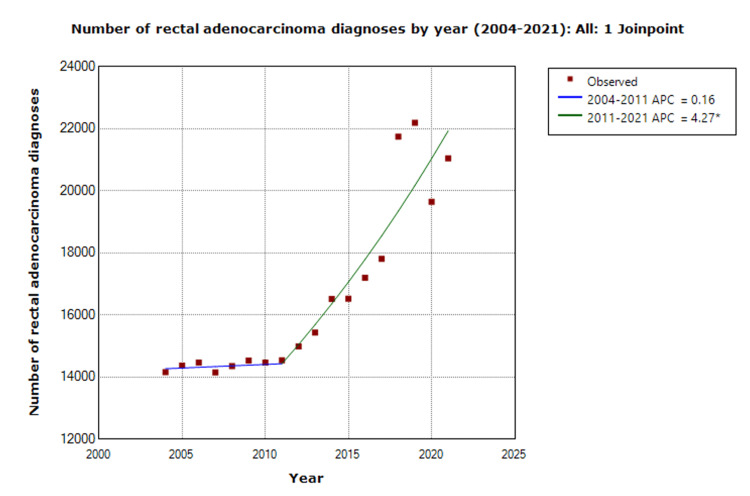
Joinpoint regression trend analysis of rectal adenocarcinoma diagnoses by year Joinpoint analysis demonstrating an increase in diagnoses of rectal adenocarcinoma over time. Note * indicates that the annual percentage change (APC) is significantly different from 0 at alpha =0.05.

Joinpoint regression analysis identified significant shifts in rectal cancer incidence trends. Between 2004 and 2011, no significant trend was observed (APC = 0.2%, p = 0.869). However, from 2011 to 2021, rectal cancer diagnoses increased significantly (APC = 4.3%, p < 0.001). Age-stratified analysis revealed that younger patients (< 50 years) experienced significant increases in the incidence of rectal cancer from 2004 to 2013 (APC = 1.9%, p = 0.004). A significantly more rapid increase in incidence was also observed in this group, with a 5.4% annual rise from 2013 to 2021 (p < 0.001). The ≥50 age group showed a non-significant decrease (APC = -0.04, p = 0.966) in rectal cancer incidence between 2004 - 2011, while from 2011-2021 a significant increase was observed in this group (APC = 4.2% increase, p < 0.001) (Figure 6, Table 8).

**Table 8:** Joinpoint regression model annual percent change (APC) estimates There is a significant increase in diagnoses of rectal adenocarcinoma from 2011 to 2021. CIL: confidence interval lower (limit); CIU: confidence interval upper (limit)

Segment	Segment start	Segment end	APC	APC 95% CIL	APC 95% CIU	p-value
0	2004	2011	0.2	-1.9	2.3	0.869
1	2011	2021	4.3	3.0	5.5	<0.001

In the propensity score matched (PSM) tumor grade analysis, median overall survival was paradoxically slightly higher among patients with poorly or undifferentiated tumors compared to those with well or moderately differentiated tumors (8.020 vs. 7.862 years), corresponding to a difference of 0.158 years (p = 0.004) (Table 9).

**Table 9:** Paradoxically reduced survival demonstrated in the poorly/un-differentiated group Our analysis revealed paradoxically reduced survival in the poorly/un-differentiated group.

	Well/moderately differentiated	Poorly/un-differentiated	Difference	p-value
Median survival (years)	7.862	8.020	0.158	0.004

On Cox proportional hazards regression analysis adjusted by PSM, poorly or undifferentiated tumor grade was associated with a modest but statistically significant reduction in the hazard of death compared to well- or moderately differentiated tumors (hazard ratio (HR) 0.954, 95% CI 0.924-0.986; p = 0.005).

## Discussion

With an increasing incidence in the younger population, rectal adenocarcinoma is becoming increasingly important to recognize and treat early on in its course. Although there is a high survival rate with local disease (91%), five-year survival rates from the SEER database remain poor for those with regional (73%) and distant spread (13%) [9]. Our study aimed to compare survival, incidence, and disease grade/stage of rectal adenocarcinoma in the United States among young and older age groups. Our analysis revealed that the overall incidence of rectal cancer increased in the period from 2011 to 2021, with the same trend seen in both individual age groups. Younger patients diagnosed with rectal adenocarcinoma presented with a higher stage and grade of disease (clinical Stages I/II and poorly or undifferentiated adenocarcinoma). In addition, Stages I and II rectal cancer had better overall survival; however, both age groups had similar survival rates. Among those with Stage III and IV disease, younger patients had worse overall survival. Lower-grade tumors had a worse median overall survival than higher-grade tumors in this study. 

Virotzko et al., in an NCBD analysis of over one million patients diagnosed with colorectal cancer from 2004 to 2015, suggested that the younger population (under 50) had more advanced disease, an increasing incidence of rectal cancer over time, and paradoxically increased survival [10]. We likewise demonstrated an increasing incidence of rectal cancer and more advanced disease in younger patients; however, we did not observe any survival benefit. Cheng et al., in a study of 769,871 individuals diagnosed with colorectal cancer (NCDB 2004-2015), and Pule et al. (South Australian Cancer Registry 2003 to 2013) both found that those over the age of 50 had worse outcomes, including all-cause mortality and disease-specific mortality, as well as short- and long-term mortality [11, 12]. This contrasts with our findings of similar mortality in early stages and increased mortality with advanced disease in younger patients. This could be due to poor screening for younger patients as opposed to older patients for whom colonoscopies are routinely recommended. With the majority presenting at later stages, it is likely that younger patients with rectal adenocarcinoma are continuing to be underdiagnosed, therefore delaying timely treatment and negatively impacting survival. The most recent guidelines from the American Cancer Society recommend colonoscopy at age 45 for average-risk patients due to the rising incidence of colorectal cancer in younger patients and modeling data that demonstrate a clear benefit [13]. This has likely led to increased detection of early-stage rectal cancer in older patients on routine colonoscopy. Our finding of no survival benefit may be due to the newer data set analyzed during this study. Our findings highlight the need for earlier screening and detection methods for rectal adenocarcinoma, as it continues to be recognized at later stages in younger patients who could significantly benefit from early aggressive therapy. The increased mortality observed with lower grades in our study may be due to multiple factors, including comorbidities in older patients (who tended to have lower-grade disease) and more aggressive treatment regimens in those with higher-grade tumors. 

As an NCBD study, our data were extracted from over 1,000 hospitals in the United States (approximately one-third of U.S. hospitals) that participate in the ACS quality improvement and surveillance initiative. Rural areas where there is less access to cancer screening and care are not included. In addition, the NCDB lacks data on recurrence, quality of life, treatment toxicity, and adherence to treatment, all of which could influence the relationship between age and outcomes in rectal cancer. Our study also did not adjust for comorbidities, socioeconomic factors, or other potential confounders. Additionally, the NCDB does not provide cause-specific survival data. As a result, our analysis is limited to overall survival, which may be influenced by non-cancer-related mortality, particularly in older patients. Our Cox variable analysis aims to control for potential confounding in the comparison between tumor grade groups. Our paradoxical findings of poorer survival in poorly and undifferentiated groups continued to be demonstrated after matching based on confounders. Future studies should aim to examine these differences amongst age groups and their effects on outcomes. In addition, the trends in rectal cancer diagnosis and incidence should be examined in a large cohort of the United States that includes those in rural areas. 

## Conclusions

Younger patients with rectal cancer often present with advanced disease and demonstrate worse outcomes than older patients when advanced, likely due to delayed diagnosis and treatment. The high prevalence of late-stage diagnoses underscores the need for improved awareness and targeted screening strategies to promote earlier detection. Addressing these disparities is essential to optimizing screening, care, and outcomes for these patients.
